# Application of a Receptor-Binding-Domain-Based Simple Immunoassay for Assessing Humoral Immunity against Emerging SARS-CoV-2 Virus Variants

**DOI:** 10.3390/biomedicines11123193

**Published:** 2023-12-01

**Authors:** Orsolya Mózner, Judit Moldvay, Kata Sára Szabó, Dorottya Vaskó, Júlia Domján, Dorottya Ács, Zoltán Ligeti, Csaba Fehér, Edit Hirsch, László Puskás, Cordula Stahl, Manfred Frey, Balázs Sarkadi

**Affiliations:** 1 Research Centre for Natural Sciences, 1117 Budapest, Hungary; mozner.orsolya@ttk.hu (O.M.);; 2 Doctoral School, Semmelweis University, 1085 Budapest, Hungary; 3 CelluVir Biotechnology Ltd., 1094 Budapest, Hungary; 4 I. Department of Pulmonology, National Korányi Institute of Pulmonology, 1121 Budapest, Hungary; 5 Department of Organic Chemistry and Technology, Faculty of Chemical Technology and Biotechnology, Budapest University of Technology and Economics, 1111 Budapest, Hungary; 6 Biorefinery Research Group, Department of Applied Biotechnology, Faculty of Chemical Technology and Biotechnology, Budapest University of Technology and Economics, 1111 Budapest, Hungary; 7 Avidin Ltd., 6726 Szeged, Hungary; 8 Steinbeis-Innovationszentrum Zellkulturtechnik, c/o University of Applied Sciences Mannheim, Paul-Wittsack-Str. 10, D-68163 Mannheim, Germany

**Keywords:** SARS-CoV-2 immunity, ELISA, spike-RBD, SARS-CoV-2 variants, Omicron, virus neutralizing

## Abstract

We have developed a simple, rapid, high-throughput RBD-based ELISA to assess the humoral immunity against emerging SARS-CoV-2 virus variants. The cDNAs of the His-tagged RBD proteins of the virus variants were stably engineered into HEK cells secreting the protein into the supernatant, and RBD purification was performed by Ni-chromatography and buffer exchange by membrane filtration. The simplified assay uses single dilutions of sera from finger-pricked native blood samples, purified RBD in 96-well plates, and a chromogenic dye for development. The results of this RBD-ELISA were confirmed to correlate with those of a commercial immunoassay measuring antibodies against the Wuhan strain, as well as direct virus neutralization assays assessing the cellular effects of the Wuhan and the Omicron (BA.5) variants. Here, we document the applicability of this ELISA to assess the variant-specific humoral immunity in vaccinated and convalescent patients, as well as to follow the time course of selective vaccination response. This simple and rapid assay, easily modified to detect humoral immunity against emerging SARS-CoV-2 virus variants, may help to assess the level of antiviral protection after vaccination or infection.

## 1. Introduction

The human immune response against viruses is based on a coordinated activation of cellular and humoral components. All factors contributing to an individual’s response to specific virus infection require complex laboratory approaches. The estimation of the T-cell-mediated antiviral cellular response requires expensive and time-consuming laboratory methodologies [1,2,3], although, recently, some simple methods, measuring the local delayed time hypersensitivity (DTH) against virus antigens applied to the skin [4], have also been advocated. Still, currently, the best-explored approach to assess the antiviral immune response is to measure the protective polyclonal antibodies generated after infection or vaccination. The key step of the SARS-CoV-2 virus infection is the entry of the virus into the epithelial cells through binding of the spike protein to its ACE2 cellular receptor [5,6]. This binding depends on the interaction of the spike receptor binding domain (RBD) with the receptor; thus, the best-known virus neutralizing effects are evoked by antibodies binding to numerous epitopes of the RBD [7,8,9,10]. Correlations of RBD-based antibody assays with virus neutralization and with real-world protection efficiency against serious disease have been widely established [11,12,13]. This is also well-exemplified by the strong clinical protective effect of monoclonal antibodies developed against the RBD protein [10,14].

A major challenge in determining the presence and effectiveness of antibodies that protect against SARS-CoV-2 is the alterations in the spike RBD, when new variants of the virus appear. It has been shown that, in immunocompromised patients undergoing antibody or plasma treatment, antibody escape variants emerge [15,16,17]. In fact, monoclonal antibodies developed against the original Wuhan-type virus [18] became ineffective against the emerging Delta, and especially the Omicron virus variants [19,20]. Thus, a relatively simple diagnostic tool to assess the virus-variant-dependent RBD binding of polyclonal antibodies generated in the human body after SARS-CoV-2 infection or immunization would be important in any further surge of this pandemic. New vaccines against emerging virus variants would require proper RBD-specific assessment of their efficiency in large populations. Also, the waning presence of such polyclonal virus neutralizing antibodies is a major problem, especially in vulnerable individuals, due to their age, underlying disease conditions, or inefficient immune response.

The SARS-CoV-2 Omicron variant has several sublineages, some of which, like the BQ.1 evolved from BA.5, have been shown to accumulate mutations that lead to lowered recognition by neutralizing antibodies [21].

The aim of the present work was to develop a simple, rapid, high-throughput methodology to study the antibody-based immunity against emerging SARS-CoV-2 virus variants and demonstrate the correlation of this assay with commercially available as well as direct virus neutralization assays. Here, we also estimated the cross-reactivity of polyclonal antibodies against the Wuhan and Omicron virus variants in the sera of vaccinated donors and convalescent patients.

## 2. Materials and Methods

### 2.1. Recombinant RBD Production

The RBD protein coding cassette with N-terminal His-tag, as in the DNA constructs presented in Amanat et al. [22], was cloned into p10 transposon vector to create stable cell lines with the SB100 Sleeping Beauty transposase. The RBD sequence was defined as the SARS-CoV-2 spike protein sequence between amino acids 319 and 541. This RBD coding sequence was modified according to mutations, resulting in amino acid changes in the examined new sublineages Omicron BA.1 (G339D, S371L, S373P, S375F, K417N, N440K, G446S, S477N, T478K, E484A, Q493R, G496S, Q498R, N501Y, Y505H), Omicron BA.5 (G339D, S371F, S373P, S375F, T376A, D405N, R408S, K417N, N440K, L452R, S477N, T478K, E484A, F486V, Q498R, N501Y, Y505H), and Omicron XBB.1.5 (G339H, R346T, L368I, S371F, S373P, S375F, T376A, D405N, R408S, K417N, N440K, V445P, G446S, N460K, S477N, T478K, E484A, F486P, F490S, Q498R, N501Y, Y505H) SARS-CoV-2 spike RBD coding sequences. The applied protein sequences are included in the Supplementary data; changes compared to the Wuhan variant are highlighted in red. The modified RBD coding DNA sequences were custom-made via gene synthesis and cloned into the plasmids used previously in the case of the Wuhan-RBD. The nucleotide sequences coding for the expressed proteins (see Supplementary Materials) have been checked by sequencing in all cases (Wuhan-RBD, Omicron BA.1-RBD, Omicron BA.5-RBD, and XBB.1.5).

For large-scale RBD protein expression, we used stable expression in HEK cells, as described previously [23], using the Sleeping Beauty transposon–transposase system and the eGFP marker protein followed by an internal ribosome entry site (IRES2) that helps to sort cells with the desired protein expression and create stable clonal cell lines (see Supplementary Materials and Supplementary Figures S1 and S2). The cell lines were grown in suspension, and the cultures were shaken (100 rpm) in serum-free media (FreeStyle 293 Expression Medium, Gibco, Cat. 12338018, Waltham, MA, USA) at 37 °C, 5% CO_2_. The RBD protein was isolated and purified by nickel ion affinity chromatography (ÄKTA pure^TM^, GE Healthcare, Chicago, IL, USA). Elution was performed by 200 mM imidazole, pH 7.4. Concentration and buffer exchange to PBS were performed by 30 kDa filters (Vivacell 100, Sartorious, Göttingen, Germany). The protein product size and purity were examined by SDS-polyacrylamide gel electrophoreses; concentration was estimated by UV–vis spectrophotometry at 280 nm. Samples were examined via Western blotting with anti-His (Sigma-Aldrich Cat.H1029, St. Louis, MO, USA) and in the case of the Wuhan-RBD anti-RBD antibody (Invitrogen Cat. MA5-38033, Thermo Fisher Scientific, Waltham, MA, USA). Anti-mouse HRP-conjugated secondary antibody (Invitrogen Cat. A16066, Thermo Fisher Scientific, Waltham, MA, USA) was used with ECL substrate for detection (BioRad Cat.1705061, Hercules, CA, USA) (see Supplementary Figure S2).

### 2.2. ELISA Method

We have determined the specific recognition of the purified proteins in their native state by the anti-RBD monoclonal primary (Abcam ab273074 and Invitrogen 703959) and HRP-conjugated secondary antibody (Abcam ab6724, Cambridge, UK) (see Supplementary Figures S2 and S3). In order to calibrate the indirect ELISA allowing for detection of RBD-specific IgG antibodies in human sera, we have applied various concentrations of the purified RBD protein and pre-COVID-19 sera, as well as sera of non-vaccinated or fully vaccinated volunteer donors. We found that 0.4 µg RBD protein and 500× dilution of the sera provided well-measurable interaction in all vaccinated donors and minimum signal in the non-vaccinated donor (see Supplementary Figure S3 and S4). In this ELISA, we used High Binding 96-well ELISA microplates (Corning Inc. Cat. 9018, Corning, NY, USA) and the tested amount of RBD in 100 µL PBS incubated overnight (16h) at 4 °C. The samples were blocked by 0.5% BSA/PBS for one hour at room temperature, then washed 3× in PBS-0.1%Tween 20. The commercially available anti-RBD monoclonal antibody (Abcam, cat. ab273074 and Invitrogen Cat.703959) was applied in 1:2000 dilution in 0.5% BSA/PBS for one hour at room temperature, washed 3 times with PBS-0.1% Tween 20. The secondary antibody (anti-rabbit HRP, Abcam, cat. ab6721) was applied in a dilution of 1:20000 in 0.5% BSA/PBS for 45 min, washed 3 times in PBS-0.1% Tween 20, and then we used TMB substrate (Thermo Scientific cat. 34021, Waltham, MA, USA) for quantification. The absorbance was read in a VictorX multilabel plate reader at 660 nm after 10 min; (A unit) was calculated by multiplying the absorbance by 1000.

In our indirect ELISA designed to measure SARS-CoV-2 spike IgG titers from human sera, we used High Binding 96-well ELISA microplates (Corning Cat. 9018, Corning, NY, USA) coated with 0.5 µg RBD in 100 µL PBS per well. Coating was performed overnight (16 h) at 4 °C. For blocking and the dilution of sera and antibodies, we used 0.5% BSA/PBS (Bovine Serum Albumin Sigma-Aldrich Cat.A7030, PBS Gibco Cat.20012027). After 1 h blocking at room temperature, we washed the plates 3 times with 0.1% Tween-20/PBS and incubated with 100 µL 1000× diluted sera in 0.5% BSA/PBS at room temperature for 1 h, washed 3 times again, and incubated for 45 min with HRP-conjugated anti-human IgG secondary antibody (Invitrogen Cat.A18805). After washing 3 times, 100 µL TMB substrate solution (TMB Substrate Kit, Thermo ScientificTM Cat. 34021 Waltham, MA, USA) per well was added; blue color reaction could be measured at 660 nm, 5 min after adding the TMB substrate; the reaction was stopped by the addition of 100 µL 0.16 M H2SO4 solution per well. The absorbance at 450 nm was measured by a Perkin Elmer Victor X3 multilabel plate reader spectrophotometer; (A unit) was calculated by multiplying the absorbance by 1000.

Each serum sample was measured in two parallels, and the average of the two measurements was calculated. Control wells without serum in the case of all three types of RBDs and controls with all used diluted serum samples with no coating antigen were used in all experiments. Sample preparation: fingertip blood samples were immediately centrifuged in polypropylene microcentrifuge tubes at 1000× *g* for 5 min, and the serum was collected from the top into a sterile new tube. Samples were then frozen and stored at −20 °C for short term and at −80 °C for long term. Clinical sera samples prepared in the hospital were stored at −80 °C. Visualization of the results was performed using GraphPad Prism 8 Software (GraphPad Software, Boston, MA, USA).

### 2.3. Virus Neutralization Assay

To produce a VSV (vesicular stomatitis virus) pseudovirus carrying the SARS-CoV-2 spike protein, codon-optimized C-terminal 17 amino acid spike gene truncations (synthesized by Geneart, Regensburg, Germany) were cloned into the eukaryotic expression plasmid pSTZ. The cloning involved the insertion of the original Wuhan spike gene (Genbank MN908947.3, Bethesda, MD, USA) containing the D614G mutation (wild-type variant), as well as the spike Omicron BA.5 variant (Genbank UPN16705.1), resulting in the recombinant plasmids pCMVspike-wt and pCMVspike-BA5. Each of these plasmids was transfected into HEK293T cells. After 24 h of transfection, the cells were inoculated with VSV-EGFP-ΔG-G (STZ Angewandte Biologische Chemie, Mannheim, Germany), a replication-deficient G-pseudotyped VSV pseudovirus encoding EGFP but lacking the genetic information for VSV-G. The inoculation lasted for one hour. Afterwards, the inoculum was removed, and the cells were washed with phosphate-buffered saline. Then, the cells were incubated in medium for 24 h. Supernatants containing the S-pseudotyped VSV-EGFP-ΔG-S pseudovirus were harvested, clarified from cellular debris through centrifugation, and stored at −80 °C. For neutralization experiments, VSV-EGFP-ΔG-S particles were preincubated for 1 h at 37 °C with varying concentrations of sera obtained from volunteer donors. The mixtures were then inoculated onto Calu-3 cells. Infection efficiency was determined at 18 h post-inoculation by fluorescence measuring using a microplate fluorescence reader (ClarioStar, BMG Labtech, Ortenberg, Germany). The neutralization data were obtained from triplicate experiments. The inhibitory concentration 50 (IC_50_) value represents the reciprocal serum dilution that results in a 50% reduction in infection. The IC_50_ data were calculated using a non-linear regression model (agonist versus response, variable slope, four parameters) with GraphPad Prism 9.0 Software.

## 3. Results and Discussion

As shown in the Supplementary Materials, Supplementary Figures S2–S4, we have confirmed that the antigenicity and recognition by neutralizing antibodies of the purified RBD protein were preserved. Moreover, in 96-well plates, a single amount of the purified RBD (0.5 μg/well) and a single dilution (1000×) of the human sera yielded satisfactory results for all the tested RBD variants to estimate the antibody levels, thus providing a one-step ELISA for anti-RBD polyclonal antibody determination.

### 3.1. Validation of the RBD-ELISA Results with Commercially Available Quantitative Immunoassay and Virus Neutralization Assay

**Figure 1:**
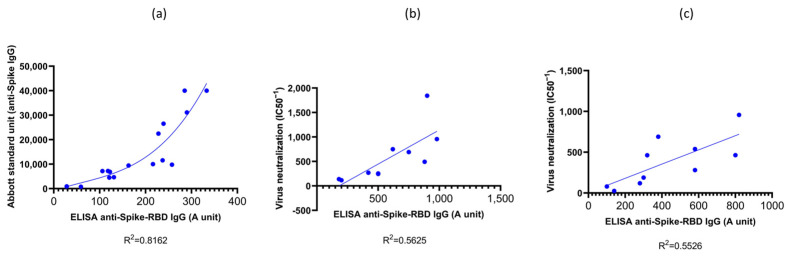
Validation of the RBD ELISA assay against a commercial immunoassay and virus neutralization assays. Panel (**a**): the Wuhan-RBD ELISA assay IgG results were compared to standard Abbott anti-spike IgG results. Panels (**b**,**c**): comparison of the Wuhan-RBD and Omicron BA.5 ELISA assay IgG results with the corresponding virus neutralization assays using the same sera samples.

First, we validated our simple indirect RBD-ELISA assay by comparing it to the quantitative Abbott SARS-CoV-2 IgG immunoassay (Abbott Alinity-I immunology analyzer # 0-AB-ALINITYI by Abbott Diagnostics, SARS-CoV-2 anti-spike IgG kit, Chicago, IL, USA) calibrated to provide standard units (SU) for the circulating IgG against the spike protein. This assay is commercially available; however, it is currently used only to measure serum IgG against the Wuhan-type SARS-CoV-2 virus variant. As shown in Figure 1a, in vaccinated individuals, we found a good correlation between the results of the two assays, especially at IgG titers above 300 SU levels. It has been shown in several publications that low anti-spike IgG levels (below 2–300 SU—corresponding to 0.1–0.2 RBD-ELISA values), although indicating an antiviral immune response or successful immunization, provide only low-level protection against real-world virus infection [20,21]. Thus, the range of antibody titers measured by the RBD-ELISA provide a good estimate of the antiviral humoral protection.

**Figure 2:**
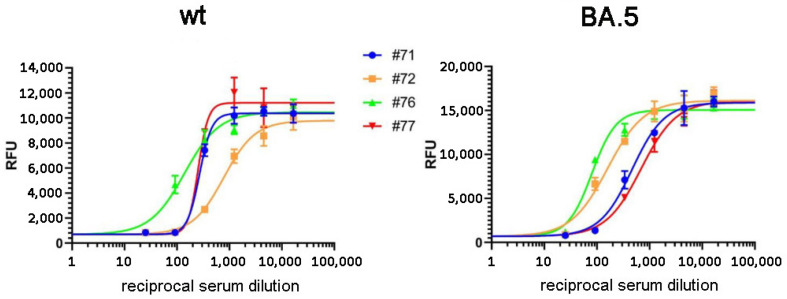
Virus neutralization assay. Neutralization curves for sera infected with Wuhan (wt) and BA.5 spike pseudotyped VSV.

In the following experiments, we validated the RBD-ELISA assay by comparing the measured anti-RBD serum IgG levels with direct virus neutralization efficiency (Figure 2). In this case, we could make this comparison both for the Wuhan-type and the Omicron BA.5 variants by using specific pseudovirus neutralization measurements. As shown in Figure 1b,c, these assays showed good correlation between the anti-RBD IgG levels and the neutralization capacity of the sera examined for both virus variants. This comparison showed that proper virus neutralization was provided only for IgG levels higher than 0.5 RBD-ELISA values, corresponding to about 300–600 SU in the commercial anti-spike IgG assay (see Figure 1a). These data indicate again that the simple RBD-ELISA assay reported here provides a valuable measure of the potential humoral defense against the SARS-CoV-2 virus variants.

### 3.2. Results of RBD-ELISA for Three Virus Variants in Vaccinated Donors and Convalescent Patients

#### 3.2.1. Results Obtained in Vaccinated Volunteer Donors

In the following experiments, we have measured the anti-SARS-CoV-2 IgG levels by using the validated RBD-ELISA assay in 45 volunteers, in parallel for all three virus variants (Wuhan, Omicron BA.1, and BA.5 variants). All individuals in this study obtained at least three vaccine shots, that is, the monovalent two-dose BNT162b2 (Pfizer-BioNTech) (commercial name: Comirnaty (Pfizer-BioNTech)) or mRNA-1273 (Moderna-NIAID) (commercial name: Spikevax (Moderna-NIAID)) mRNA vaccine doses, and at least one booster shot (either homologous or heterologous forms of these vaccines). The time period after the last vaccination was between 3 and 12 months. These measurements were made easy by using only a few µL of sera from fingertip pricking, without the need for venous blood drawing.

**Figure 3:**
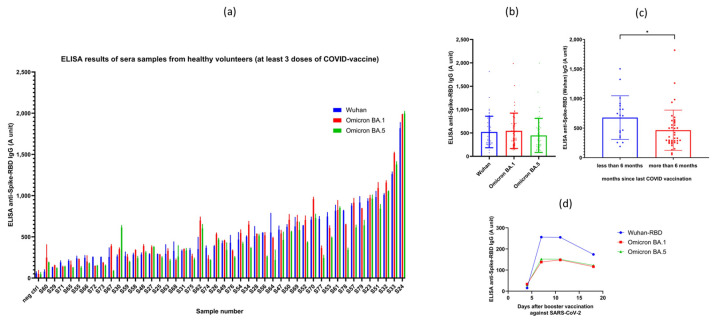
RBD-ELISA results obtained in vaccinated volunteers. IgG antibody titers (A unit) are shown against the Wuhan, Omicron BA.1, and Omicron BA.5 RBDs. (**a**) IgG levels against the three examined variants in vaccinated volunteers (a serum sample collected before the COVID-19 pandemic was used as negative control), (**b**) IgG level results grouped by variants, (**c**), IgG levels measured against the Wuhan-RBD grouped by the time period after booster COVID-19 vaccination, and (**d**) measurement of the IgG titers of an individual who received the 4th COVID-19 vaccine at time 0.

As shown in Figure 3a, all fully vaccinated individuals had well-measurable anti-RBD antibody titers, although the range of these titers was very wide, from 0.2 to 2.2 units. Interestingly, all vaccinated individuals, although at variable levels, also had antibodies against the Omicron variants. Since all these volunteers were vaccinated by the original Spikevax (Moderna-NIAID) or Comirnaty (Pfizer-BioNTech) vaccines, containing RNA coding for the spike protein of the Wuhan variant, this was somewhat unexpected, although already noted in the relevant literature [24,25,26]. While some of the volunteers may have been infected with the SARS-CoV-2 Omicron variant(s) in question, this is unlikely in this healthy volunteer cohort (see volunteer data).

Figure 3b shows collected data for the antibody titers against the three virus variants examined. Partly due to the relatively high scattering of the data, we found no statistical difference in the recognition of the RBD variants in the vaccinated individuals, although in many cases the Omicron BA.5 variant was less efficiently recognized in these volunteers. Supplementary Figure S5 shows how, in the case of the Omicron XBB.1.5 variant, this recognition decreased compared to the Wuhan-RBD. Figure 3c shows the effect of the time period after the last booster shot obtained in the vaccinated individuals. There is a significant decrease in the anti-RBD IgG levels after the longer time period following the last vaccination.

The one-step RBD-ELISA from finger-pricked serum samples against the virus variants enables following the specific IgG levels on a daily basis, without the burden of taking venous blood samples. Indeed, as shown in Figure 3d, after a booster shot, the relatively low anti-RBD levels in this individual were greatly increased at about the 6th day following this re-vaccination. Interestingly, the Comirnaty (Pfizer-BioNTech) mRNA vaccine against the original SARS-CoV-2 spike protein significantly increased the anti-RBD IgG levels against the Omicron variants as well. Based on these experiments, we suggest that our simple ELISA method may be an important tool to assess the effects of multiple vaccinations in immunocompromised individuals, or with diseases affecting a proper immune response after vaccination.

#### 3.2.2. Results Obtained in Convalescent Patients after COVID-19 Disease

In this study, we have analyzed the anti-RBD polyclonal IgG levels in 21 patients, treated in the National Korányi Institute of Pulmonology in Hungary, in the period of January 2020–November 2021, with various manifestations of the COVID-19 disease: mild cases: patient recovered at home, without hospital treatment; moderate: patient recovered after hospital treatment, but not at the intensive care unit; severe: patient recovered from illness after hospital treatment at the intensive care unit; critical: patient recovered from illness after hospital treatment at the intensive care unit, with mechanical ventilation.

The collected sera after convalescence (within 7 days after recovery from illness) were stored at −80 °C and analyzed by our RBD-ELISA for all three SARS-CoV-2 variants. During the time these sera were collected, the Omicron virus variants were not present in Hungary. Consequently, these results indicate a cross-reactivity of the antibodies generated in the convalescent patients against the examined virus variants.

**Figure 4:**
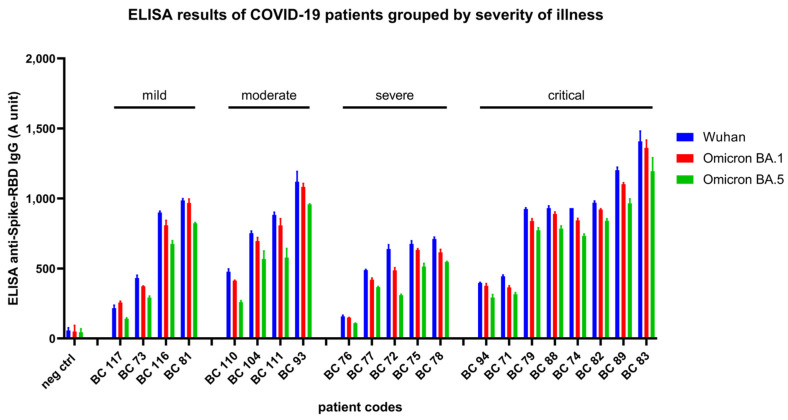
Anti-RBD (Wuhan, Omicron BA.1, and BA.5) ELISA IgG titers of COIVD-19 patients grouped by severity of illness. A serum sample collected before the COVID-19 pandemic was used as negative control.

As shown in Figure 4, in all these patients, we found a significant level of anti-RBD polyclonal antibodies for all three SARS-CoV-2 variants. Interestingly, the clinical severity (mild, moderate, severe, or critical level—see Supplementary Figure S3) of COVID-19 disease did not correlate with the antibody titers; that is, lower and higher levels of anti-RBD antibodies were found in each group of patients. Although there is a tendency for higher anti-RBD IgG levels in patients with COVID-19 disease at a critical level, the number of patients examined here is not enough to provide a quantitation of this potential difference.

## 4. Conclusions

As a summary, here, we document the applicability of a simple RBD-based ELISA to analyze the humoral immune response, that is, anti-RBD IgG titers in vaccinated or convalescent patients. The sera obtained from small finger-pricked blood samples enable wide and easy sample collection, the assay uses a single dilution of the serum sample, and the assay can be performed in any commercially available ELISA developer and reader system. The simplified preparation of the RBD protein of emerging virus variants enables rapid adjustment of the assay to any new surge of the disease.

An interesting finding of the present work is that, although to a highly variable extent, protective antibody production against the original Wuhan variant also coincides with the emergence of protective antibodies against two Omicron variants of the SARS-CoV-2 virus, both in vaccinated donors and in convalescent patients. In any further emerging SARS-CoV-2 variants, such a rapid and simple assay may help to screen the actual humoral immune protection, which has been demonstrated to undergo changes with recent variants [27]. We also demonstrated the application of this ELISA in the case of the recent Omicron XBB.1.5 variant (see Supplementary Figure S5). We suggest that the presented and validated assay, easily modified to be applied in the case of newly emerging virus variants, may be helpful in a population-wide screening or a daily follow-up of potential antiviral humoral protection.

## Data Availability

The data presented in this study are available on request from the corresponding author.

## Supplementary Materials

The following supporting information can be downloaded at: https://www.mdpi.com/article/10.3390/biomedicines11123193/s1, Figure S1: p10-RBD-IRES2-EGFP vector map; Figure S2: RBD protein production; Figure S3: ELISA with different serum dilutions grouped by variants; Figure S4: RBD IgG ELISA results with different serum dilutions (Wuhan, BA.1, and BA.5 variants) grouped by dilution; Figure S5: RBD (Wuhan and Omicron XBB.1.5 variant) IgG titers in sera samples from healthy volunteers and recovered COVID-19 patients. Supplementary data: protein sequences of the produced RBD proteins (Wuhan, Omicron BA.1, BA.5, XBB.1.5).
