# Supplementary material for: Application of a Receptor-Binding-Domain-Based Simple Immunoassay for Assessing Humoral Immunity against Emerging SARS-CoV-2 Virus Variants
Source: Biomedicines. 2023 Dec 1;11(12):3193. doi: 10.3390/biomedicines11123193 (PMC10740953; doi:10.3390/biomedicines11123193)
Supplement: Supplementary file 1 [file biomedicines-11-03193-s001.zip › biomedicines-2708941-supplementary.pdf]

Supplementary materials for Mózner et al. “Application of an RBD-based simple immunoassay for assessing humoral immunity against emerging SARS-CoV-2 virus variants”

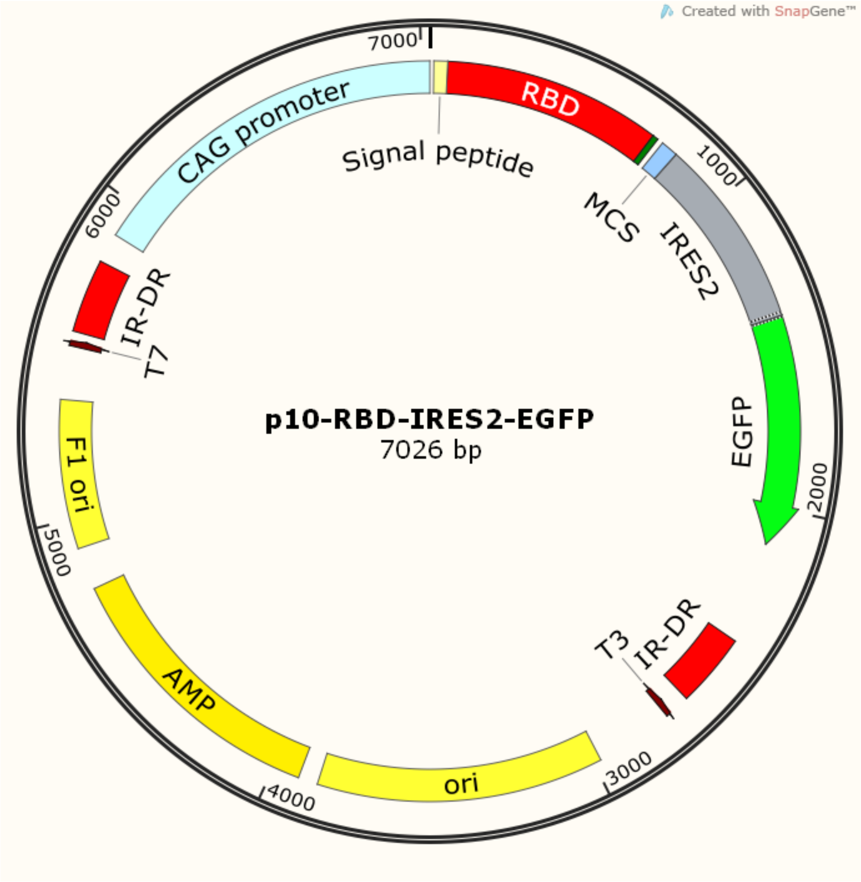

Suppl. Figure S1. The p10-RBD-IRES2-EGFP transposon vector map

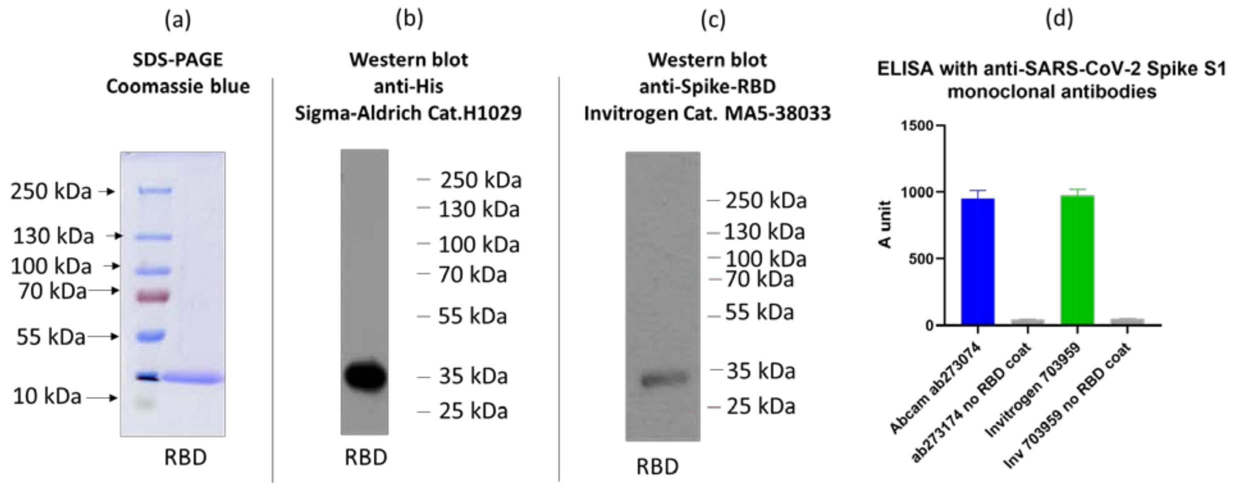

Suppl. Figure S2. The RBD production was examined by SDS-polyacrylamide gel electrophoreses and Western blotting. (a) Shows the purified RBD protein after SDS-PAGE and Coomassie blue staining. (b) Shows the purified RBD protein after SDS-PAGE and western blot using the anti-His primary antibody (Sigma-Aldrich Cat.H1029). (c) Shows the purified RBD protein after SDS-PAGE and western blot using the anti-RBD primary antibody (Invitrogen Cat. MA5-38033). (d) ELISA results of RBD(Wuhan) detected by two anti-SARS-CoV-2 Spike S1 commercially available monoclonal antibodies (Abcam Cat. ab273074 and Invtitrogen Cat. 703959), controls: ELISA coating without RBD.

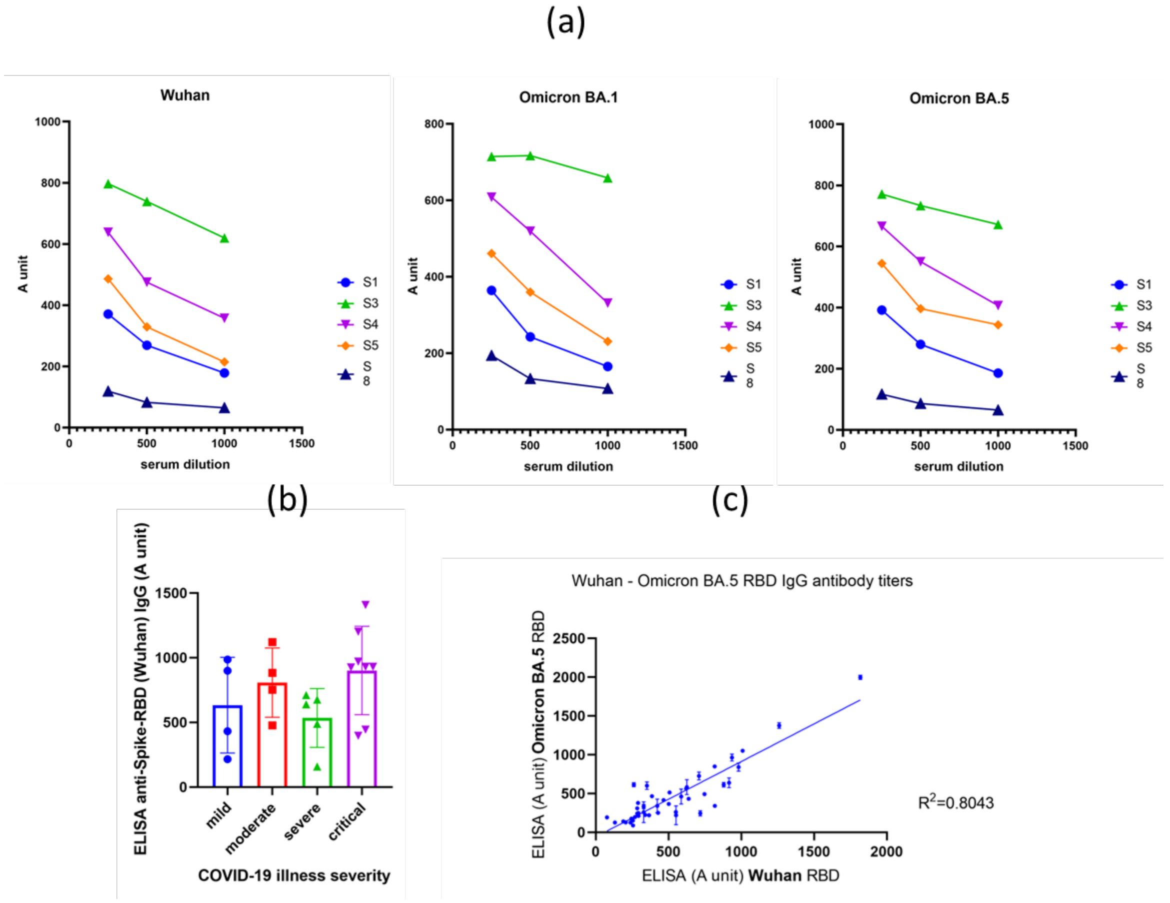

Supplementary Figure S3. (a) Anti-RBD (Wuhan, Omicron BA.1 and Omicron BA.5 RBD) IgG antibody titers - ELISA results of 5 individuals (serum dilutions 250x, 500x, 1000x) samples gathered in 11/2022. S1, S3, S4, S5 are serum samples of fully vaccinated individuals, while S8 is a sample of a non-vaccinated individual. (b) Anti-RBD (Wuhan) IgG antibody titers of 21 recovered COVID-19 patients grouped by severity of illness. (c) Linear regression of IgG antibody titers against Wuhan and Omicron BA.5 RBDs measured in volunteers.

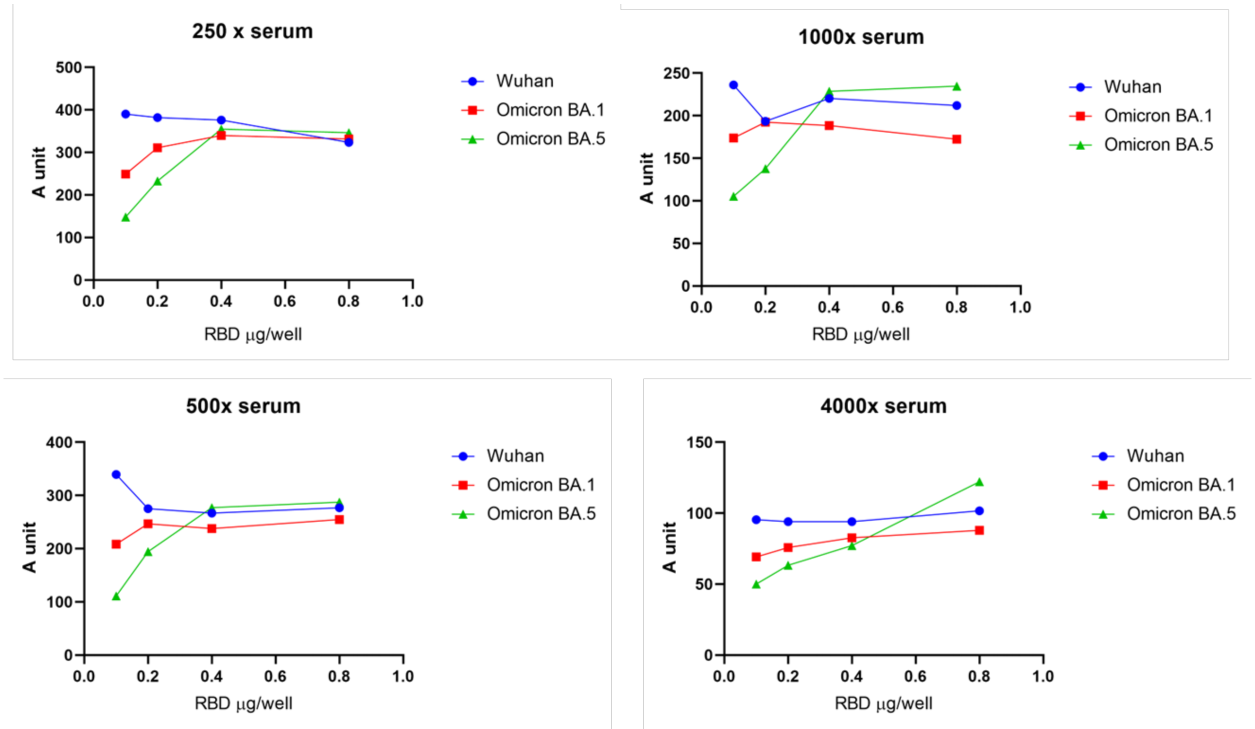

Supplementary Figure S4. ELISA measurement with different RBD protein amounts and serum dilutions of a fully vaccinated individual, sample gathered in 11/2022. Absorbance measured at 660 nm10 minutes after addition of TMB substrate.

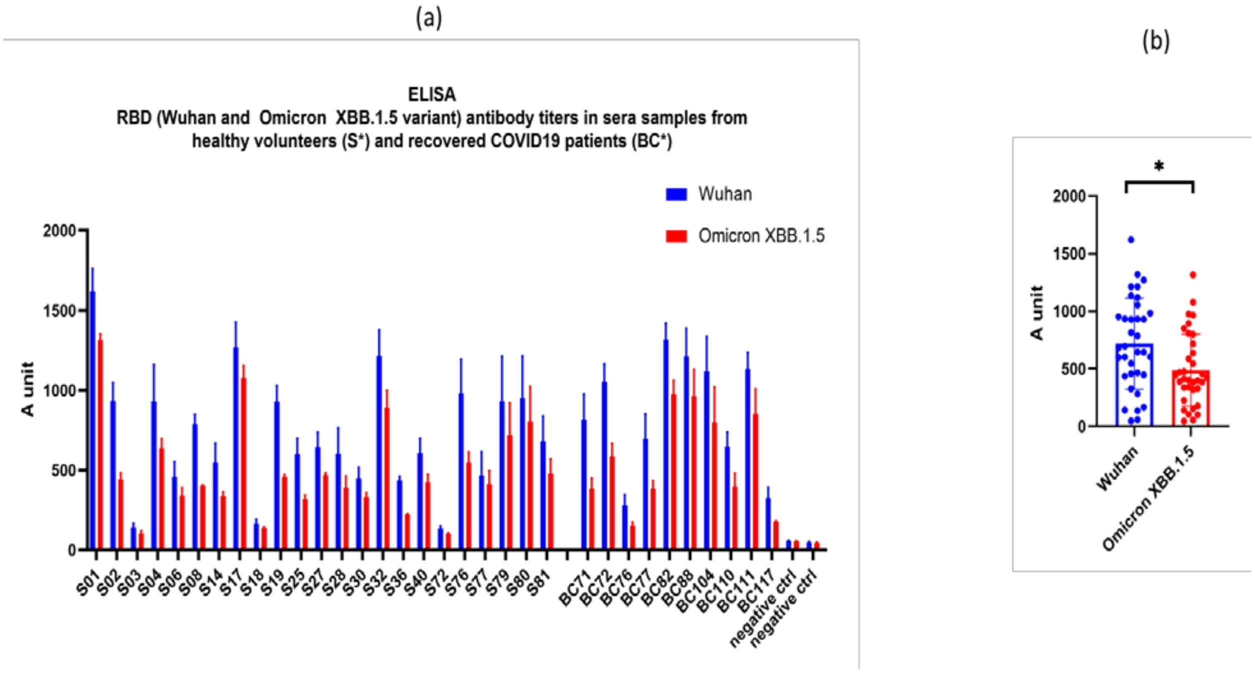

Supplementary Figure S5. ELISA measurement. (a) Anti-RBD (Wuhan and Omicron XBB.1.5) antibody titers in vaccinated (S*) and recovered individuals (BC*). (b) Summary: antibody titer means of anti-Wuhan-RBD and anti-Omicron XBB.1.5.

Supplementary data:

RBD (Wuhan) protein sequence (319-541 of SARS-CoV-2 Spike):

MFVFLVLLPLVSSQRVQPTESIVRFPNITNLCPFGEVFNATRFASVYAWNRKRISNCVADYSVLYNSASFSTFKCYGVSPT
KLNDLCFTNVYADSFVIRGDEVRQIAPGQTGKIADYNYKLPDDFTGCVIAWNSNNLDSKVGGNYNYLYRLFRKSNLKPF
ERDISTEIYQAGSTPCNGVEGFNCYFPLQSYGFQPTNGVGYQPYRVVVLSFELLHAPATVCGPKKSTNLVKNKCVNFHH
HHHH*

RBD (Omicron BA.1)

MFVFLVLLPLVSSQRVQPTESIVRFPNITNLCPFDEVFNATRFASVYAWNRKRISNCVADYSVLYNLAPFFTFKCYGVSPT
KLNDLCFTNVYADSFVIRGDEVRQIAPGQTGNIADYNYKLPDDFTGCVIAWNSNKLDSKVSGNYNYLYRLFRKSNLKPFE
RDISTEIYQAGNKPCNGVAGFNCYFPLRSYSFRPTYGVGHQPYRVVVLSFELLHAPATVCGPKKSTNLVKNKCVNFHHH
HHH*

RBD (Omicron BA.5 – same as Omicron BA.4**)**

MFVFLVLLPLVSSQRVQPTESIVRFPNITNLCPFDEVFNATRFASVYAWNRKRISNCVADYSVLYNFAPFFAFKCYGVSPT
KLNDLCFTNVYADSFVIRGNEVSQIAPGQTGNIADYNYKLPDDFTGCVIAWNSNKLDSKVGGNYNYRYRLFRKSNLKPF
ERDISTEIYQAGNKPCNGVAGVNCYFPLQSYGFRPTYGVGHQPYRVVVLSFELLHAPATVCGPKKSTNLVKNKCVNFHH
HHHH*

RBD (Omicron XBB.1.5)

MFVFLVLLPLVSSQRVQPTESIVRFPNITNLCPFHEVFNATTFASVYAWNRKRISNCVADYSVIYNFAPFFAFKCYGVSPT
KLNDLCFTNVYADSFVIRGNEVSQIAPGQTGNIADYNYKLPDDFTGCVIAWNSNKLDSKPSGNYNYLYRLFRKSKLKPFE
RDISTEIYQAGNKPCNGVAGPNCYSPLQSYGFRPTYGVGHQPYRVVVLSFELLHAPATVCGPKKSTNLVKNKCVNFHHH
HHH*
